# Developmental Status: Impact of Short-Term Ischemia on Follicular Survival of Whole Ovarian Transplantation in a Rabbit Model

**DOI:** 10.1371/journal.pone.0135049

**Published:** 2015-08-13

**Authors:** Shuangshuang Xie, Xing Zhang, Wenming Chen, Chichi Xie, Wenwei Chen, Pu Cheng, Ying Zhou, Bicheng Chen

**Affiliations:** 1 Obstetrical Department, the Second Affiliated Hospital of Wenzhou Medical University, Wenzhou, Zhejiang, 325000, China; 2 Centre for Reproductive Medicine, the First Affiliated Hospital of Wenzhou Medical University, Wenzhou, Zhejiang, 325000, China; 3 Surgical Laboratory, the First Affiliated Hospital of Wenzhou Medical University, Wenzhou, Zhejiang, 325000, China; 4 Transplantation Centre, the First Affiliated Hospital of Wenzhou Medical University, Wenzhou, Zhejiang, 325000, China; 5 Oncological Surgery, the Second Affiliated Hospital of Zhejiang University School of Medcine, Hangzhou, Zhejiang, 310000, China; Centro Cardiologico Monzino, ITALY

## Abstract

Ischemia is the first mechanism that provokes the loss of follicles in ovarian grafts over the long term. In whole ovarian transplantation, it remains unknown, however, how changes in follicular development are influenced by short-term ischemia. Fresh whole ovarian orthotopic auto-transplantation was performed in rabbits with 45 min ischemia, and the impact of ischemia on follicular survival and development status was evaluated at different time-points (1 day, 3 days, 1 week, 2 weeks and 1 month). Assessment of follicular quantity and morphology was carried out via histologic analysis. Follicle proliferating status was evidenced by immunostaining with proliferating cell nuclear antigen (PCNA), and the Hedgehog signaling pathway (Patched and Gli); was verified via TUNEL assay. Quantitative PCR was carried out to quantify the mRNA of target genes including PCNA, Patched, Gli, Caspase 3, Bax, and Bcl-2. Compared with its contralateral fresh controls, the morphology, proliferation and apoptosis of the follicles in the grafts showed no significant differences and most primordial follicles were quiescent. However, morphology and proliferation status were significantly decreased 1 week after grafting, in comparison with the longitudinal grafting time. Patched and Gli in the Hedgehog signaling pathway were activated in only the follicles of the grafts. Short-term ischemia slightly impacts follicular survival and development status in whole ovarian grafting. Receiving intervention in the first week post-transplantation might be helpful.

## Introduction

Aggressive chemotherapy/radiotherapy and bone marrow transplantation can cure > 90% of girls and young women affected by disorders requiring such treatment[1]. However, the ovaries are very sensitive to those treatments [2–4]. Therefore, preserving patients’ reproductive function remains one of the most pressing issues in this area. Several options are currently available to preserve fertility, including oocyte, fertilized embryo and ovarian tissue preservation. Hitherto, there have been twenty-four live births by ovarian tissue orthotopic transplantation worldwide [1]. Whole ovarian transplantation represents an exciting new technique that can improve follicular pool maintenance and avoid follicle depletion. Compared to ovarian cortex transplantation, whole ovary transplantation through vascular anastomosis would reduce the warm ischemic time, which will most likely improve follicle survival [5,6]. The first report of a successful transplantation with follicle development in humans was in the year 2008, in a patient who had complete orthotopic grafting of fresh whole ovaries to treat Turner’s syndrome[7]. Although this seems to be an achievement for a promising treatment, as a result of the grafting, the possibility of follicle reserve depletion still exists and thus limits fertility restoration.

Evidence from reimplantation of frozen-thawed whole ovaries has shown that there are two primary significant mechanisms that provoke follicle loss. The first mechanism is ischemia [1,6,8], which is an inevitable event prior to cryopreservation. A previous animal study demonstrated that 65% of the follicles were lost after fresh tissue grafting; adding cryopreservation and thawing increased follicle loss by only another 7% [8]. Meanwhile, after fresh reimplantation, two mechanisms are responsible for this follicular loss: 1) ischemia caused injury and delayed reoxygenation, and 2) follicular activation [1]. Ischemia is the most significant factor overall that caused follicular loss. The second mechanism is also the result of ischemic injury mainly affected by poor vascular bed preparation.

The mechanisms behind ischemic injury involve energy depletion and reperfusion oxidative stress, which produces reactive oxygen species (ROS), such as hydroxyl radicals, superoxide anion, and hydrogen peroxide (H2O2) [9–11].This can eventually cause damage to lipids, DNA, enzymes and structural proteins, leading to cell death [12,13]. Meanwhile, gene expression of several inflammatory factors is initiated by hypoxia-sensitive response elements, resulting in the transmigration of neutrophils and macrophages into the tissue that causes tissue destruction and fibrosis [6]. Therefore, keeping ischemia time to a minimum is crucial and urgent during the transplantation procedure. To increase surviving follicles, research should focus on overcoming ischemia injury.

Furthermore, significant progress has been made in understanding graft regulation and recovery of ovarian functionality under the influence of ischemia. However, the early stages of whole ovarian grafting on ovarian activity (follicular development) have largely been unstudied. Exploring the influence of ischemia at the early stage of ovarian transplantation may improve the understanding of how to promote the recovery of ovary functionality and ultimately offer guidance for intervention. In the present study, we aim to determine the impact of short-term ischemia on the survival and development of follicles at the early stages (within 1 month) of fresh ovarian auto-transplantation in a rabbit model. To evaluate the effects of ischemia on follicular development in a whole ovary graft, expression levels of proliferating cell nuclear antigen (PCNA), Hedgehog signaling pathway members (Patched and Gli), Bcl-2 family members (Bcl-2 and Bax) and Caspase3 mRNA, in addition to TUNEL assay and histological examination, were compared between grafts and controls at several critical developmental time points.

## Materials and Methods

### Animals

All experiments were conducted in accordance with the Guide for the Care and Use of Laboratory Animals published by the U.S. National Institute of Health (NIH Publication No. 85–23, revised 1996). All animal studies were reviewed and approved by the ethics committee of Wenzhou Medical University, China. A total of 20 sexually mature, 5-month-old female Japanese white rabbits with body weights of 2.0 to 2.5 kg were used. They were housed in the animal center of Wenzhou Medical University in a temperature-controlled environment (23±2°C) with 12 h light/12 h dark cycles. Food and water were available ad libitum.

### Whole ovary transplantation

**Fig 1:**
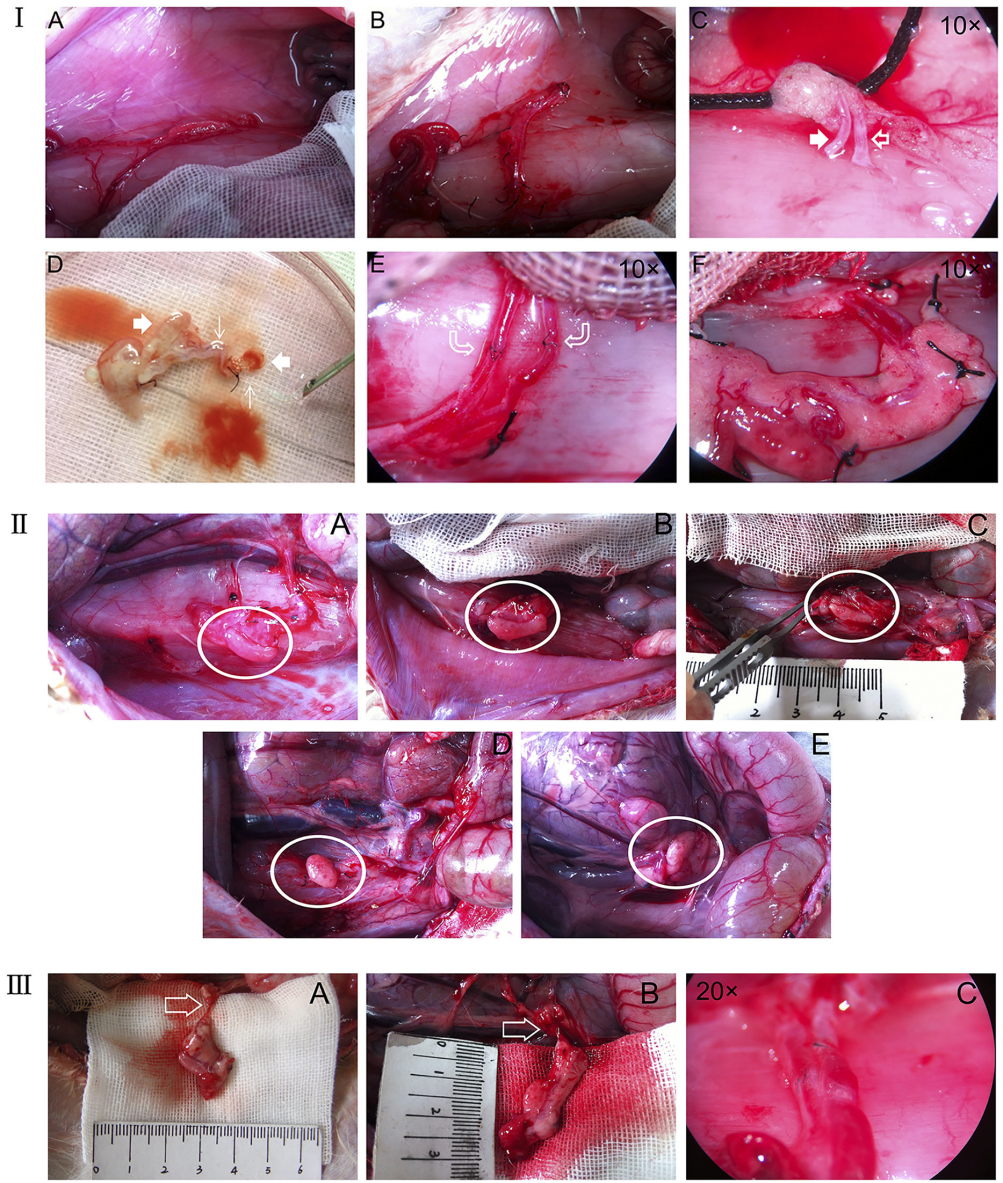
Observation of vessels and ovary during the grafting period. (I. A-F) State of ovarian artery and vein at the end of the anastomosis. (II. A-E) State of ovary (white circle) at different grafting time-points (1 day, 3 days, 1 week, 2 weeks, and 1 month, respectively). (III. A-C) State of ovarian artery and vein 1 month after grafting.

After anesthesia with diethyl ether and 1.5% pentobarbital sodium (1 ml/kg), each rabbit underwent a midline laparotomy and bilateral ovarian oophorectomy. The right pedicle stump was then dissected and incised for anastomosis. Only the left whole ovary and its supplying vessels that were 2.5 cm away from the ovarian hilum were used for transplantation. The right whole ovary was saved as a fresh control (0 day). After the left ovarian artery (diameter range: 0.3–0.5 mm) and vein (diameter range: 0.9–1.5 mm) were isolated, they were immediately cannulated with a 30-gauge straw needle in an artery (internal diameter: 0.2–0.4 mm). The ovaries were then submerged in and gently perfused with pre-cooled 100 IU/mL of heparin sodium saline for 15 min until clear liquid exited the ovarian vein. After perfusion, the left intact ovary was immediately contralaterally autografted via microvascular end-to-end anastomosis to the right pedicle stump. The ovarian artery was joined by six interrupted stitches with 11–0 nylon suture (JinHuan Medical Products; China) under ×20 magnification. Subsequently, the ovarian vein was joined by continuous stitches with 10–0 nylon suture (JinHuan Medical Products; China) under ×10 magnification (Fig 1I). The entire transplantation procedure was completed in approximately 30 min. Ischemia time was defined as the time between ovary removal and vessel unclamping and was limited to 45 min. Blood circulation was confirmed via observation of a reddish ovarian cortex and pulsation through the anastomosed vessels within 30 min postoperatively. One injection of heparin sodium (500 U/kg) was given after surgery. Twice-daily injections of gentamicin (× 1 ml) were given for 3 consecutive days, starting on the day of transplantation. Observation of vital signs was performed once a day.

### Ovarian tissue collection and treatment

The autografts were removed at time-points of 1 day (n = 4), 3 days (n = 4), 1 week (n = 4), 2 weeks (n = 4), or 1 month (n = 3 because one rabbit died as a result of infection) after transplantation. Then, rabbits were euthanized with an intravenous injection of sodium pentobarbital (100 mg/kg).

Recording the macroscopic appearance of the ovaries and making particular note of peri-ovarian adhesions was necessary (Fig 1II). For this study, every contralateral fresh ovary (0 day, which is not grafting and directly preserved) was pre-processed once after being removed; one-half was fixed in 4% paraformaldehyde for histological examination and immunochemistry, the other was placed in -80°C and prepared for subsequent mRNA quantification. The treatment of each grafting ovary after removal was the same as for the contralateral fresh ovary. Afterward, fixation for the grafts and controls was completed with 4% paraformaldehyde; the grafts and controls were continually sectioned into two pieces of equal size (a quarter into the ovary) and then were embedded in paraffin. Beyond a quarter into the ovary, each graft ovary was sliced in ten serial 3-μm sections and five slices were randomly selected. Overall, a total of 30 fields were selected to evaluate the follicular healthy rate (HR), PCNA+ rate, and TUNEL+ rate of each ovary.

### Follicle counts

With hematoxylin-eosin stain, the number of total morphologically normal follicles (MNF), MNF of different levels, and total follicles present were all recorded. To avoid double counting follicles, only follicles with a visible oocyte nucleolus were counted. MNF were identified as follicles with normal nuclei and homogeneous cytoplasm surrounded by well-organized granulosa cells. In contrast, follicles were considered degenerated when one or more of the following features were observed: pyknotic oocyte nuclei, an empty space in the oocyte cytoplasm, or granulosa cell degeneration [14]. Follicle counts were obtained in a high-power field (×400), including 3 developmental stages as primordial follicles, primary follicles and secondary follicles. The healthy rate (HR) is defined as the number of MNF in a certain developmental stage divided by the number of total follicles in the same developmental stage. The total healthy rate (THR) represents the total number of MNF divided by the total number of follicles. Atretic follicles were not included in any count. The difference value (D-value) was calculated using the following formula: D-value = THR (contralateral control)—THR (graft).

### Immunohistochemistry of PCNA

After deparaffinization and rehydration, slices were treated with 0.3% H_2_O_2_ for 10 min at room temperature to inactivate endogenous peroxidase activity, and then sodium citrate was used to retrieve the epitopes. Afterward, slices were incubated with 5% normal goat serum for 30 min at 37°C to block nonspecific staining. The slices were incubated overnight at 4°C with anti-PCNA antibodies (1:100; Abcam; USA). Then, the slices were incubated for 30 min at 37°C with a secondary antibody (ZSGB-BIO; China). 3,3N-Diaminobenzidine Tertrahydrochloride (DAB) was added to change the color by incubating the slices for 2 min and counterstaining with hematoxylin for 3 min. Only the granulosa cells stained red–brown in color in the nucleus were specified as positive (otherwise, the cells were considered to be negative) [15]. Each stage of positively stained follicles and the total number of each stage of follicles was counted in a high-power field (×400). The ratio of each stage of PCNA+ follicles to the total number of each stage of follicles was calculated.

### Immunofluorescence analysis of Patched and Gli

For immunofluorescence, the slices with tissue were treated according to standard procedures. The ovary slices were double-immunofluorescence stained with mouse monoclonal antibody Patched (1:100; Origene; USA) and goat polyclonal antibody Gli (1:100; Santa Cruz; USA) on the same slice and the slices were stored at 4°C overnight. Sequentially, slices were incubated with donkey anti-mouse IgG and donkey anti-goat IgG (1:200; Earrhox; USA). Then, slices were processed with DAPI staining. Double-immunofluorescence was assessed utilizing a microscope (Leica CH-9435; Germany) and images of follicles on the slices were recorded using LAS software at wavelengths of 591 nm for Patched staining and 493 nm for Gli staining. Follicles with red and green highlighted areas in the cytoplasm of granulosa cells or oocytes were called Patched positive, and Gli positive, respectively [16].

### TUNEL assay

A terminal deoxynucleotidyltransferase-mediated dUTP nick end labeling (TUNEL) assay was used to assess apoptosis. A commercial kit (In Situ Cell Death Detection Kit, Roche; Switzerland) was used in accordance to the manufacturer’s instructions. At the same time, the negative and positive controls were set and the macroscopic appearance of the apoptotic ovarian cells was photographed; the number of positive cells was recorded. Follicles with red-brown coloring of the nucleus in the granulosa cells were designated as positively stained (otherwise, as negative staining). The positively stained cells and the total number of cells were counted under ×200 magnification. The ratio of TUNEL+ cells to the total number of cells was calculated.

### Quantification mRNA of target genes

Frozen tissue samples were prepared for mRNA extraction using liquid nitrogen and Trizol Reagent (Ambion 28218; Life Technology; USA). Single-strand cDNA was synthesized from 1-μg high-quality mRNA using the ReverTra Ace qPCR RT Kit (Toyobo; Osaka, Japan). The final 20-μL reaction mixture was incubated at 37°C for 15 minutes for reverse transcription. The reaction was inactivated by heating at 98°C for 5 minutes.

PCNA, Patched, Gli, Caspase3 and Bax/Bcl-2 gene expression were semi-quantified via real-time fluorescent PCR (F-PCR) using the QuantiFast SYBR Green PCR (Qiagen Inc. Germany). cDNA amplification was performed with PCR primers (S1 Table). The PCR condition used was the same for all primer sets; the amplification program consisted of an initial activation step at 95°C for 5 minutes followed by 40 cycles of 10 s denaturation at 95°C and 30s annealing and extension at 60°C. Standards and samples were amplified in duplicate and each gene expression level was normalized by consulting a 2^-△△CT^ method [17].

### Statistical analysis

Experimental data are presented as the means ± SEM and analyzed by SPSS 17 (IBM SPSS software, US, Chicago). Comparisons of the D-value of total healthy rate (THR) and mRNA quantification of target genes (2^-△△CT^) among the grafts were calculated using a Kruskal-Wallis H test. Comparisons of the D-value of total healthy rate (THR) and mRNA quantification of target genes (2^-△△CT^) among the grafts were calculated using a Kruskal-Wallis H test. Comparisons of the healthy rate (HR) and number of follicles positive for PCNA at each follicle level between grafts and contralateral fresh controls were evaluated using a Wilcoxon signed rank test. The proportions of follicles positive for PCNA and TUNEL among grafts were compared via X^2^ analysis, and Fisher’s exact test was applied when the expected value was <5. For all statistical analyses, *P*<0.05 was considered to be significant.

### Animal ethics

This study was carried out in strict accordance with the recommendations in the Guide for the Care and Use of Laboratory Animals of the National Institutes of Health. The protocol was approved by the Committee on the Ethics of Animal Experiments of Wenzhou Medical University (Permit Number: WYDW2012-0037). All surgery was performed under sodium pentobarbital anesthesia, and all efforts were made to minimize suffering.

## Results

### Increased difference value of the follicle healthy rate

**Fig 2:**
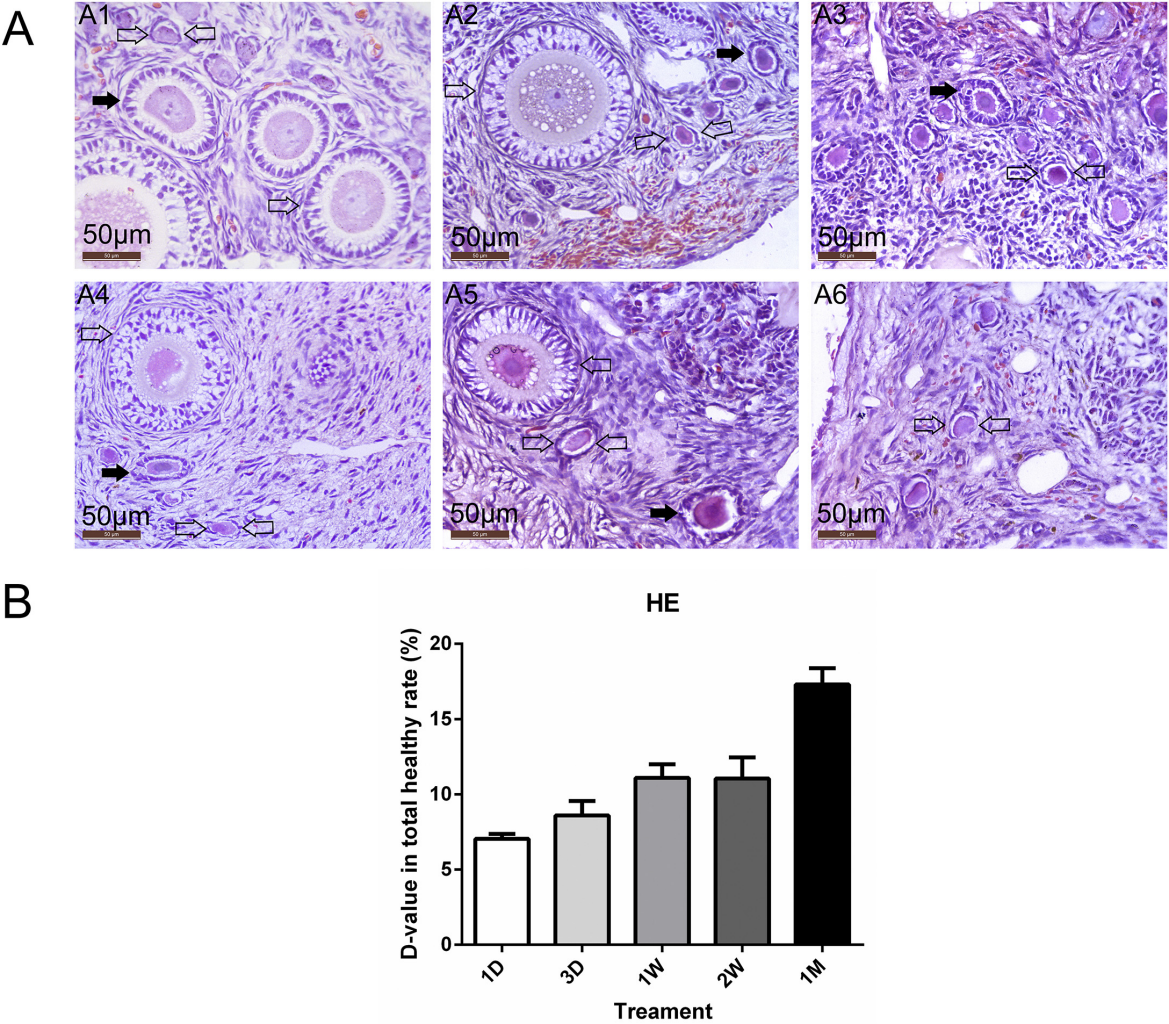
Morphology of follicles in grafts at different time-points. (A) Histological examination of ovaries. Distribution of morphologically normal follicles in each developmental stage was identified, including primordial (bi-directional arrow), primary (filled arrow) and secondary follicles (open arrow). (A1) fresh control. (A2-6) ovaries after grafting (1 day, 3 days, 1 week, 2 weeks, and 1 month, respectively). Original magnification ×400. Scale bar = 50 μm. (B) Differences in follicular total healthy rate (D-value of THR) among grafts showed a significantly increased trend when grafting time increased. 1D = 1 day, 3D = 3 days, 1W = 1 week, 2W = 2 weeks, 1M = 1 month.

Nineteen animals in each time-point group (including 19 fresh controls and 19 grafts) were analyzed for both structural organization of the ovary and the stages of follicles that were present. Representative images of MNF in each time-point group are shown in Fig 2A, where the distribution of follicles in each developmental stage was identified, including primordial, primary, secondary and antral follicles. The total healthy rate (THR) and healthy rate (HR) of each follicle level in the grafts were not significantly different from contralateral fresh ovaries at each time-point (*P*>0.05); however, the D-value at different time-points showed an increased trend according to the grafting time (*P*<0.05; Fig 2B). The D-value in the 1 day group was significantly lower than in the 1 month group (7.05±0.65 vs. 17.30±1.87, *P*<0.05; Table 1). Moreover, there was no difference between 1 day and 3 days for the D-value, nor 1 week and 2 weeks (*P*>0.05).

**Table 1:** The total rate of healthy follicles and differences in values of total healthy rate.

Group	G-S (%)	C-F (%)	P-value	Differences (%)
**1 Day**	71.13±3.91	78.18±3.82	NS	7.08±0.61*
**3 Days**	68.98±3.77	77.58±2.97	NS	8.60±1.92*
**1 Week**	61.95±2.82	73.05±3.53	NS	11.10±1.79**
**2 Weeks**	65.30±3.43	76.35±1.44	NS	11.05±2.80**
**1 Month**	62.53±1.96	79.83±2.93	NS	17.30±1.87

Note: G-S, grafting groups; C-F, contralateral fresh.

Values are means ± SD.

NS = not significant.

* means significant difference compared to the 1 week, 2 weeks and 1 month time points (*P* <0.05).

* *means significant difference compared to the 1 month time point (*P* <0.05).

### Alternation expression of PCNA

**Fig 3:**
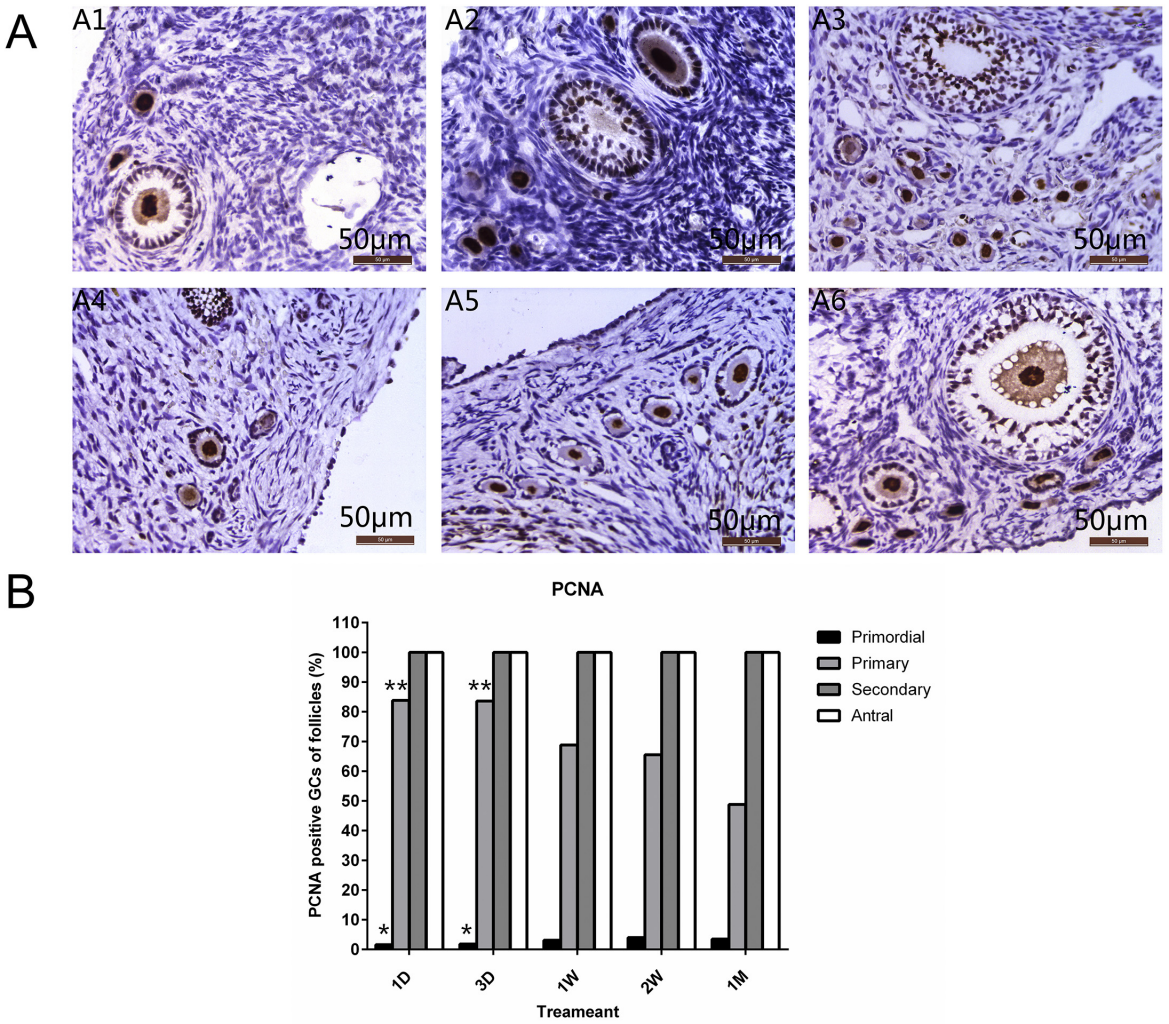
Proliferation of follicles in grafts at different time-points. (A) Immunohistochemical staining for the cell proliferation marker, PCNA, in the transplanted ovaries. Red-brown coloring of the nucleus of the granulosa cells was recorded as positive staining (bi-directional arrow). (A1) fresh control. (A2-6) ovaries after grafting (1 day, 3 days, 1 week, 2 weeks, and 1 month, respectively). Original magnification: ×400. Scale bar = 50 μm. (B) The proportion of PCNA-positive primordial, primary, secondary and antral follicles for various time-point grafts. PCNA-positive rate of primordial and primary follicles, respectively, showed significantly increased and decreased trends when grafting time increased. * means significant difference compared to the 1 week, 2 weeks and 1 month time points (*P* <0.05). * *means significant difference compared to the 1 month time point (*P* <0.05) 1D = 1 day, 3D = 3 days, 1W = 1 week, 2W = 2 weeks, 1M = 1 month.

Follicles were immunostained with PCNA, an antigen expressed in the nuclei of cells during the DNA synthesis phase of the cell cycle, to compare the proliferative status at different timepoints, as illustrated in Fig 3A; PCNA identifies the positive granulosa cells in the primordial and primary follicles. There was no significant difference between the controls and grafts for the proportion of total PCNA+ in primordial and primary follicles (*P*>0.05; Table 2).

**Table 2:** PCNA in primordial follicles and primary follicles.

**Follicle**	Proportion of PCNA-positive follicles (%) (positive follicles/follicles evaluated)									
Stage	1 Day		3 Days		1 Week		2 Weeks		1 Month	
	G-S	C-F	G-S	C-F	G-S	C-F	G-S	C-F	G-S	C-F
**Primordial**	1.57%*	7.00%	1.84%*	5.20%	3.10%	11.10%	4.00%	11.10%	3.50%	9%
	(22/1403)	(16/228)	(26/1420)	(54/1038)	(18/590)	(24/216)	(26/657)	(48/432)	(23/655)	(36/400)
**Primary**	83.30%*	90.10%	83.60%*	91.00%	68.80%	77.10%	65.50%	73.40%	48.80%	92.90%
	(83/99)	(128/142)	(51/61)	(48/53)	(53/77)	(64/83)	(55/84)	(72/98)	(41/84)	(52/56)

Note: G-S, grafting groups; C- F, contralateral fresh

* means significant difference compared to the 1 week, 2 weeks and 1 month time points (*P* <0.05).

As shown in Fig 3B and Table 2, a high proportion of primordial follicles was quiescent as indicated by PCNA negative at all timepoints; a high proportion of primary follicles were active, and thus, were positive. A similar proportion of primordial and primary follicles showed PCNA+ granulosa cells in grafts both at 1 day and 3 days (*P*>0.05); from Day 7, a significantly higher proportion of PCNA+ primordial follicles and significantly lower proportion of PCNA+ primary follicles were found than at 1 day and 3 days in the grafts (*P*<0.05). No significant differences of either PCNA + primordial or primary follicles were found among 1 week, 2 weeks, and 1 month. The secondary and antral follicles were excluded from statistical comparison in all granulosa cells labeled with PCNA in secondary and antral follicles in the controls and grafts.

**Fig 4:**
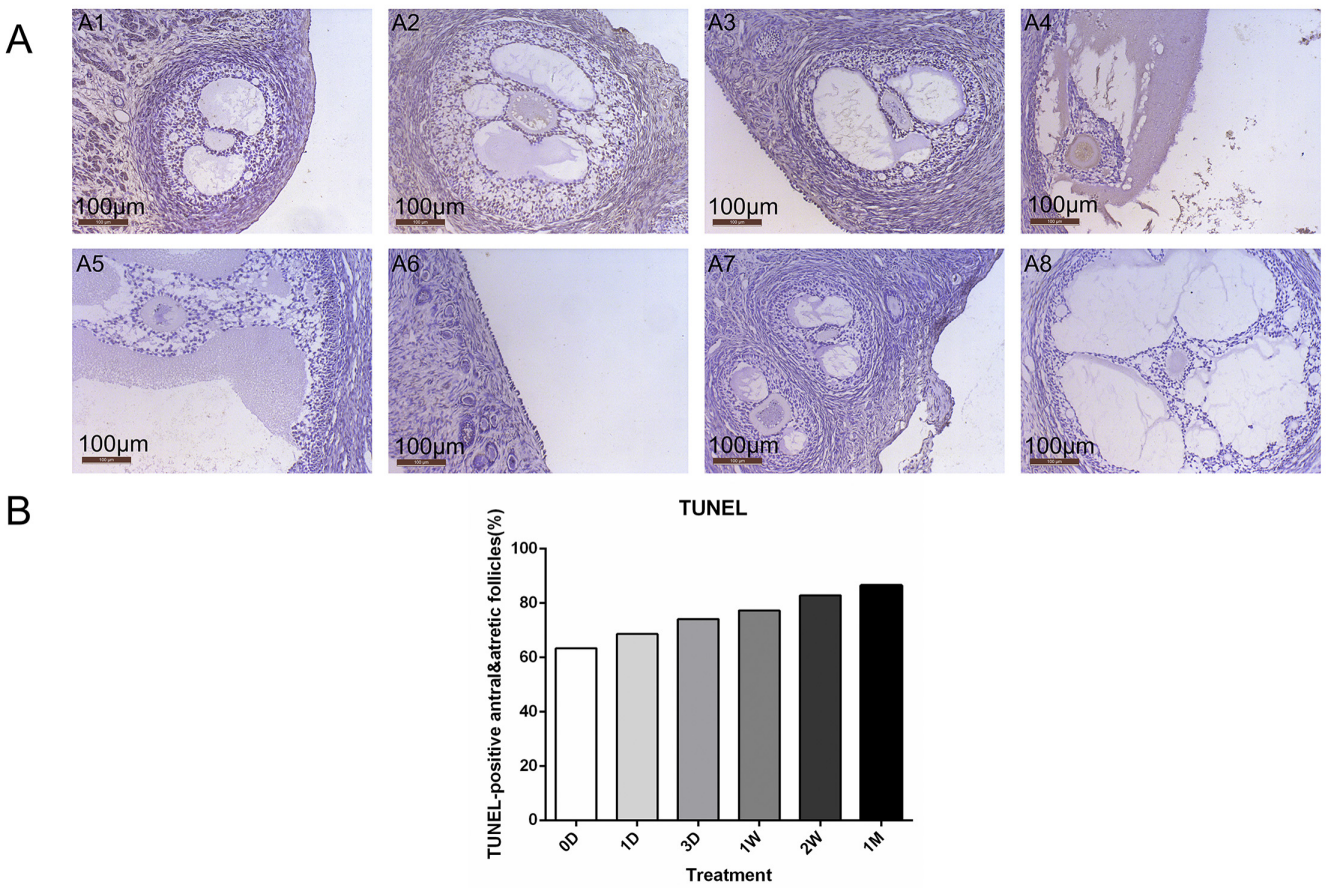
Quantification of target gene mRNA. (A) The expression of PCNA mRNA varied in grafting groups showing a decreased trend following grafting time (*P* <??). (B) Expression of Patched and Gli mRNA (*P*>0.05). (C-D) Expression of Caspase-3 mRNA and Bax:Bcl-2 ratio (*P*>0.05). * means significant difference (*P* <0.05). NS: not significant.

The transplantation process caused a significant down-regulation of the expression of PCNA mRNA (*P*<0.05; Fig 4); however, there was no significant difference between 1 day and 3 days (*P*>0.05), which was consistent with immunocytochemistry.

### Similar apoptosis rate

**Fig 5:**
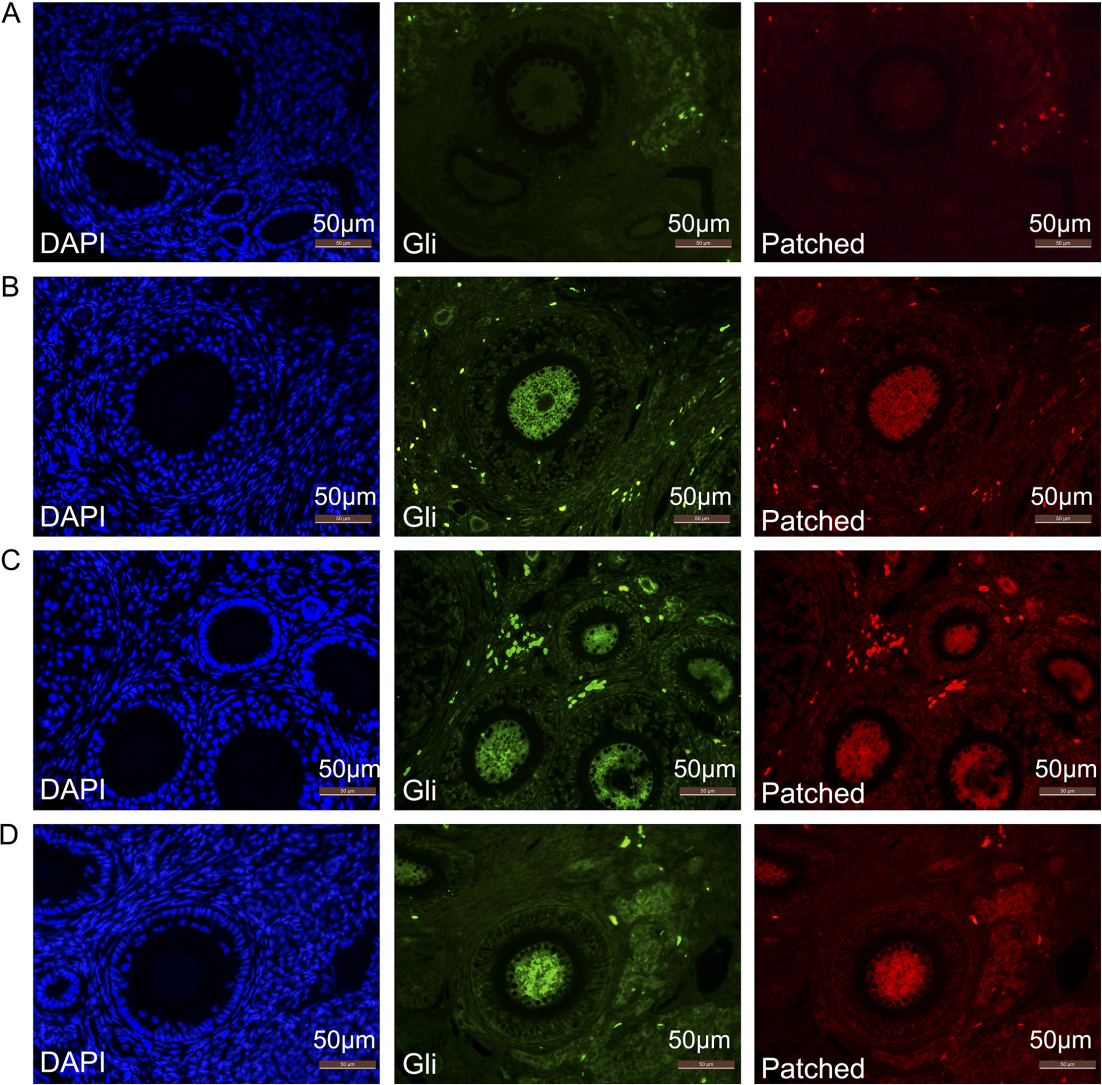
Identification of the apoptosis in grafts at different time-points. (A) TUNEL assay for grafted ovaries. Red-brown coloring was recorded as positive staining mainly in antral and atretic follicles. (A1-4) TUNEL staining of fresh controls, grafting at 1 week, 2 weeks and 1 month, respectively. (A5-8) Negative control. Original magnification: ×200. Scale bar = 100 μm. (B) TUNEL-positive rates among different timepoints were not found to be significantly different following auto-transplant in grafts (*P*>0.05). NS: not significant. 1D = 1 day, 3D = 3 days, 1W = 1 week, 2W = 2 weeks, 1M = 1 month.

Unexpectedly, most follicles were negative in the TUNEL assay in ovarian tissue from the controls (1,244 follicles), 1 day (789 follicles), 3 days (1,006 follicles), 1 week (624 follicles), 2 weeks (693 follicles), and 1 month (654 follicles). The TUNEL+ cells were mainly found in antral follicles and early atretic follicles, which were both with TUNEL+ granulosa cells along the antral border (Fig 5A). Occasionally, small isolated follicles with granulosa cells stained positive for TUNEL. Therefore, only antral follicles and early atretic follicles were included in the count and comparison. A total of 30 antral and early atretic follicles were found in fresh controls, 35 follicles in 1 day’s grafts, 27 follicles in 3 day’s grafts, 22 follicles in 1 week’s grafts, 35 follicles in 2 weeks’ grafts, and 37 follicles in 1 month’s grafts. The proportions of TUNEL+ follicles were not found to be significantly different within each time-point (*P*>0.05; Table 3).

**Table 3:** TUNEL staining of antral and atretic follicles.

Follicle stage	TUNEL-positive follicles (%, positive follicles/ follicles evaluated)					
	0 Day	1 Day	3 Days	1 Week	2 Weeks	1 Month
**Antral and atretic follicles#**	63.30%	68.60%	74.10%	77.30%	82.90%	86.50%
	(19/30)	(24/35)	(20/27)	(17/22)	(29/35)	(32/37)

# No significant difference was found among the timepoints (*P*>0.05, X^2^ test).

Real-time PCR showed that auto-transplantation did not result in differences of apoptosis associated gene expression in ovary tissue among the grafts, including the Caspase3 and Bax:Bcl-2 ratio (*P*>0.05; Fig 4).

### Activation of hedgehog signaling

**Fig 6:**
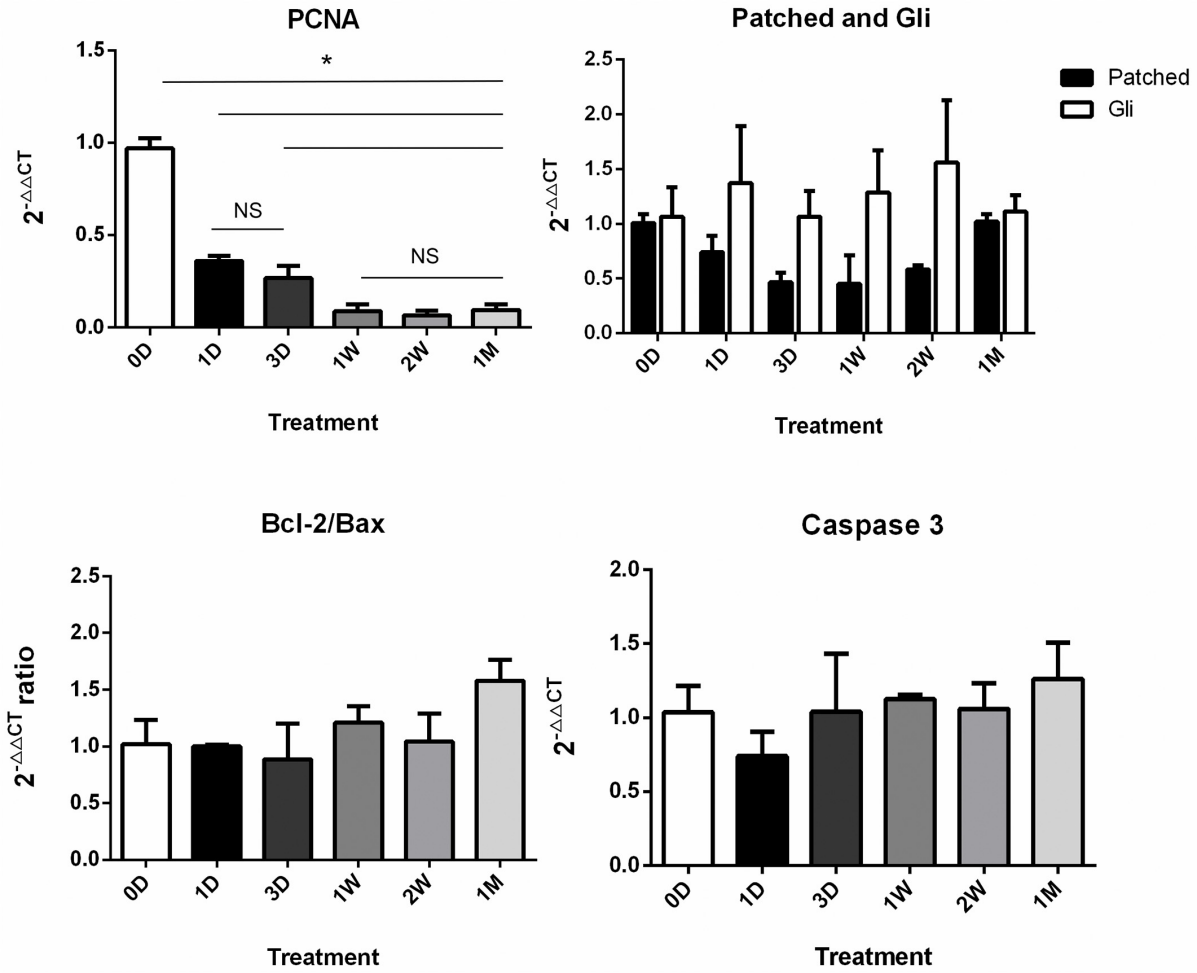
Immunofluorescence staining of Patched and Gli. Immunostaining for Patched (red color) and Gli (green color) was observed in the cytoplasm of granulosa cells or oocytes of primordial, primary, secondary and antral follicles; no positive mark for the fresh control group. (A) Fresh controls. (B) Ovaries after grafting for 3 days. (C) Ovaries after grafting for 1 week. (D) Ovaries after grafting for 1 month. Original magnification: ×400. Scale bar = 50 μm.

As illustrated in Fig 6, immunostaining for Patched and Gli in grafts was observed in the cytoplasm of granulosa cells or oocytes of primordial, primary, secondary and antral follicles, ranging from faint to strong in intensity. Meanwhile, signal intensity of Patched and Gli was found to be similar following grafting at everygrafts. However, no signal was observed in the fresh controls (0D). However, there were no significant differences in mRNA expression of Gli and Patched among the grafts from different time-points (0D, 1D, 3D, 1W, 2W, 1M) (*P*>0.05).c.

## Discussion

In the present study, we systematically investigated the impact of short-term ischemia on follicular survival and development status following whole ovary orthotopic autotransplantation at the early stages post-transplantation (within 1 month). The results indicated that follicular survival and development status were slightly influenced by ischemia during the observation period. With 45 min ischemia, the THR, HR of each follicle level, PCNA expression in primordial and primary follicles, and apoptosis in antral and early atretic follicles were similar to those in contralateral fresh ovaries at every time-point. Interestingly, most of the primordial follicles remaining at 1 month are quiescent during 1 month. However, in comparison with the longitudinal grafting time, the HR of follicles and the number of PCNA+ follicles in primary follicles gradually and inevitably decreased 1 month after grafting. Moreover, an essential promotional pathway, the Hh pathway, was found in all growing grafts.

Ischemia is the main factor behind follicular pathogenesis and depletion in grafts rather than cryopreservation in ovarian transplantation [8]. Transplant operation experience and skills and the methods chosen determined the ischemia time. We controlled the vascular anastomosis operative time strictly to within 30 min and limited ischemia time to 45 min. Both in practice and in theory, orthotopic grafting with an end-to-end anastomosis enables immediate revascularization and avoids thrombogenesis, as shown in our study. For our preliminary heterotopic model, including 30 rabbit fresh ovaries with vascular anastomoses in the carotid artery and the jugular vein, all failed from a closed vessel (data not shown). Functioning vascularization is crucial for the survival of whole ovary transplantation, providing immediate and sustained revascularization of the ovary tissue. In the present study, all levels of ovarian blood vessels are patent and full of erythrocytes at the medulla or cortex of the grafted ovaries.

It is generally accepted that early follicular development, which is regulated by ovarian autocrine/paracrine regulators, is closely related to oocyte-granulosa cells-ovarian stromal cells/theca cells [18]. The growth and development of ovarian follicles is characterized by marked processes of granulosa cell proliferation and differentiation, such as PCNA [19]. In a previous animal study, PCNA staining was absent from granulosa cells and oocytes of the quiescent primordial follicles, but was intensive in most granulosa cells and oocytes in primary-to-large antral follicles [20]. The presentation in our study was consistent with these results. Further, the proportion of PCNA+ of primordial and primary follicles was similar between the controls and grafts. Activation of resting primordial follicles is a strictly regulated process that appears to be disrupted by grafting [21]. Thus, our results may indicate that follicular survival was slightly influenced by short-term ischemia, and follicular development status may be well preserved at a minimum.

Furthermore, PCNA and TUNEL tests are not always correlated with morphological changes. Therefore, a comprehensive analysis of these phenomena in follicles should incorporate morphotic cell evaluations [22]. TUNEL assay supports the identification of ovarian cells with the characteristic symptoms of apoptosis, including those in early stages of degeneration, when morphological changes sometimes are not pronounced[23]. In the current study, we found that THR and HR at each follicle level in the grafts were similar to those in the contralateral fresh controls, which was consistent with the PCNA immunochemistry results. Meanwhile, the proportion of TUNEL positive observed in only antral and early atretic follicles also showed no significant difference between the fresh and controls. Meanwhile, apoptosis associated gene expression in ovary tissue, including Caspase3 and Bax: Bcl-2 ratio, showed no significant differences among the groups. All of the results above co-indicate that follicular survival was slightly influenced by ischemia in whole ovarian transplantation within 1 month. The very low proportion of PCNA+ and lack of TUNEL+ observed in primordial follicles suggested that they were in a quiescent status and can survive for a relatively long time period after suffering short-term ischemia. A likely reason is that they have very low metabolic demands and thus are less sensitive to ischemic stress. Additionally, because they are distributed just under the ovarian surface, they may be benefit from additional oxygen from the ingrowth of new peri-ovary vessels [6]. Moreover, in line with previous studies, apoptosis was mostly found in antral and atretic follicles, but not in primordial follicles[24–26].

It is critical to maintain the quantity and activity of follicles in the process of ovarian tissue transplantation. Follicular quantity is thought to be one of the best indices for evaluating ovarian function. In the current study, significant changes of D-value and PCNA+ rate in primary follicles from Day 7 indicated that follicular quantity and activity gradually and inevitably decreased and degenerated following grafting time and suggested that the first week post-transplantation may be the crucial time-point for ovarian activity and function. The mRNA level of PCNA from the grafts agreed with this result. There may be some damages to lipids, DNA, enzymes and structural proteins, which were caused by schemic injury and reperfusion injury, leading to cell death eventually[9–13]

Ovarian follicle formation and development involve remodeling events that are regulated by families of developmental signaling pathways, including hedgehog (Hh) [27]. The Hh signaling pathway is critical for ovarian function in Drosophila, and over the last decade, the mammalian ovary has been the subject of research [16,28–31]. The Hh pathway regulates embryonic development as well as the function of adult tissues through effects on cell proliferation, differentiation, and survival [32]. In the absence of ligand binding, Patched maintains Hh in an inactive state. When one of the ligands binds to Patched, signaling occurs through the downstream transcription factors, Gli [27,33–35]. In the current study, follicular proliferation status was additionally demonstrated by Patched and Gli. Except for fresh controls, Patched and Gli expression were observed in all stages of follicles in the grafts, indicating probable activation of the Hh pathway in follicles after grafting. However, there were no significant differences in mRNA expression of Gli and Patched among the grafts from different time-points (0D, 1D, 3D, 1W, 2W, 1M) (*P*>0.05). Regulation of the mRNA in transcription process or post-transcription among the process of gene expression may be the reasonable explanation for this result.

The expression of Patched and Gli was also not consistent with PCNA+ in primary follicles, which suggested that Hh activation did not only reflect proliferation. It has been demonstrated that the up-regulated expression of the Hh-signaling cascade in a Gli–dependent or independent way was resistant to inflammation in ischemia tissues, such as in heart disease, cholestatic liver or liver injury [36–38]. The Hh pathway induces neovascularization and up-regulates vascular endothelial growth factor (VEGF) and angiopoietins [39] while a new supply of oxygen and nutrients is being created. This evidence suggested that Hh may be a protective pathway that is activated after ischemic injury in whole ovary transplantation.

This study is the first to demonstrate that short-term ischemia in a rabbit whole–ovary auto-transplantation model slightly impacted follicular survival with good evidence of follicular activity after 1 month. The Hh signaling pathway was significantly involved in the ischemia response. Although a significant number of degenerated and apoptotic follicles was not detected, follicular loss was gradually observed, implying that early intervention may reduce the loss of the follicular population over the long term. In the future, based on the current study of fresh whole ovary, further studies for early- and long-term survival of follicles should be conducted and combined with cryopreservation such as slow-freezing or vitrification. Studies for some interventions such as antioxidant or anti-apoptotic agents should also be taken into account. More studies are required to access the mechanisms behind activation of the Hh signaling pathway in ischemia of whole ovarian grafting and its impact on follicular survival. Although whole-ovary freezing and transplantation has not yet enjoyed the same success as ovarian cortical tissue freezing and transplantation, and cancer patient will at risk of thrombus of whole-ovary transplantation, it is still technically possible and pending further investigation, supports the use of this model system for human studies to restore endocrine activity and fertility.

## Supporting Information

S1 Table Primer sequences used in real-time fluorescence PCR. (DOCX)
