# Supplementary material for: Developmental Status: Impact of Short-Term Ischemia on Follicular Survival of Whole Ovarian Transplantation in a Rabbit Model
Source: PLoS One. 2015 Aug 13;10(8):e0135049. doi: 10.1371/journal.pone.0135049 (PMC4536045; doi:10.1371/journal.pone.0135049)
Supplement: S1 Table — (DOCX) [file pone.0135049.s001.docx]

**Table S1 Primer sequences used in real-time fluorescence PCR**

| **Gene** | **Forward Primer (5´—3´)** | **Reverse Primer (5´—3´)** |
| --- | --- | --- |
| **PCNA (XM_002710838.1)** | GAGAACTGGGAAATGGAAACAT | ACTGTAGGAGAAAGCGGAGTG |
| **GLI (XM_002720910.1)** | AGCCAGAGAGACCAGCAACT | TCCGACAGAGGTGAGATAGACA |
| **Patched(XM_002708260.1 )** | AGATTGGGGAAGAGGCTATGTT | GAGTCCAGGTGTTGAAGGAGTG |
| **Caspase3(NM_001082117.1 )** | CGTGAAGAAATCATGGAACTCA | ACCGTGGCTTAGAATCACACA |
| **Bcl-2 (DQ529234.1)** | CCCAGGGACAGCATATCAGA | GCTCCAGGTGGTCATTCAGG |
| **Bax (XM_002723696.1)** | CGTTGTCGCCCTGTTTTACT | TCCAGCCCATAATAGTCCTGAT |
